# Systemic Lupus Erythematosus in Shwachman-Diamond Syndrome: a Novel Phenotype

**DOI:** 10.1007/s10875-022-01425-z

**Published:** 2023-01-04

**Authors:** Tianyu Zhang, Zhongxun Yu, Sihao Gao, Lin Wang, Hongmei Song

**Affiliations:** Department of Pediatrics, Peking Union Medical College Hospital, Chinese Academy of Medical Sciences & Peking Union Medical College, Beijing, 100730 China

To the Editor:

Originally, Shwachman-Diamond syndrome (SDS) is recognized as a genetic disorder with classical clinical presentations of exocrine pancreatic dysfunction, skeletal abnormalities, and bone marrow dysfunction mainly due to mutations in the Shwachman-Bodian-Diamond Syndrome gene (*SBDS*) on chromosome 7. Accumulating data supports that immunological disorders can occur in SDS patients. Here, we described an SDS patient initially presented with systemic lupus erythematosus (SLE), which had not been reported elsewhere.

A 17-year-old Chinese female who was diagnosed and treated as SLE by another hospital came to our department complaining of recurrent headaches. We carefully reviewed the medical history and found that she was born to healthy non-consanguineous parents at full term without complications. Childhood vaccinations, growth, and development were all normal. However, at the age of 5 months, she started to experience recurrent respiratory infections and mild leukopenia/neutropenia. She experienced two episodes of left upper extremity fractures at the age of 8 and 11, respectively.

At 15 years old, she developed eyelid edema 3 days after respiratory infection and edema of both lower limbs 1 month later. She did not seek medical advice until 9 months later when she developed gaze palsy, salivation, and loss of consciousness for 3 min. After regaining consciousness, she developed headache, dizziness, abnormal gait, and blurred vision and then was admitted by a local hospital. Neurological examinations revealed drunk gait, limited range of abduction of the right eye, and abnormal heel-to-shin test. Laboratory examinations showed leukopenia (3.1 × 10^9^/L), neutropenia (0.9 × 10^9^/L), proteinuria (3 + , 24- h urine protein 2.4 g), hypocomplementemia (C3 0.496 g/L, C4 0.035 g/L), and positive autoantibodies [ANA(+)H1:1000, anti-dsDNA and anti-SSA/Ro antibodies(+), ACL(+)]. Her IgM was slightly lower than normal (0.376 g/L), while her hemoglobin, platelets, lymphocyte subset counts, thyroid function, and electrolyte were normal. Chest CT showed a few small patchy opacities in the right lower lobe, indicating inflammation (Fig. 1a). Video EEG suggested background asymmetry with slow wave activity in the right occipital area. Head MRI showed multiple abnormal signals in the pons, cerebellum, and medulla, indicating inflammatory lesions (Fig. 1b, c). Head MRA and MRV were normal, and cerebral thrombosis was discarded. A lumbar puncture was performed during the acute phase, and the pressure was measured about 100 drops/min. The cerebrospinal fluid examination disclosed a mild CSF pleocytosis (39 cells/uL, 2.6% neutrophils, 97.4% monocytes) with normal glucose, protein, and chloride. Microbial PCR, cultures, and autoimmune panel results were all negative. But she did not perform a renal biopsy. She was diagnosed as SLE with involvement of hematological, renal, and neuropsychiatric systems. She received intravenous methylprednisolone 0.5 g for 3 days for 2 rounds followed by prednisone 60 mg QD and cyclophosphamide (CTX) 1 g every 3 weeks. Most lab data turned normal except her leukocytopenia and ANA status. The prednisone was tapered accordingly, and the CTX was switched to mycophenolate mofetil (MMF) after 9 rounds of treatment. Unfortunately, at 16 years old, she developed pain and lameness of lower extremities, and her hip MRI indicated osteonecrosis of the bilateral femoral head, which was considered as side effects of long-term use of corticosteroids.

**Fig. 1:**
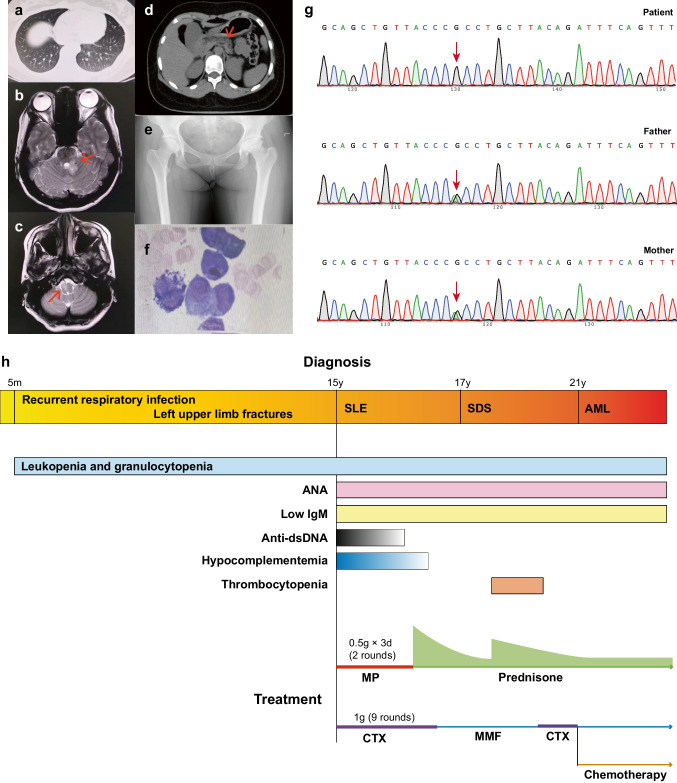
Examinations and the timeline of clinical management. **a** Chest CT showed a few small patchy opacities. **b, c** Multiple abnormal signals in brain MRI. **d** Shrunken pancreas with fat infiltration. **e** Osteoporosis of the right hip joint and avascular necrosis of the left femoral head. **f** Bone marrow aspirate supporting the diagnosis of acute myeloid leukemia (AML-M2). **g** Sanger sequencing results of *SBDS *from the indicated family members. **h** Timeline of clinical management of this case. ANA, antinuclear antibodies; AML, acute myeloid leukemia; CTX, cyclophosphomide; MMF, mycophenolate mofetil; MP, methylprednisolone; SDS, Shwachman-Diamond syndrome; SLE, systemic lupus erythematosus

After carefully reviewing all of her above medical history, we agreed her diagnosis as SLE but still wondered why she had non-organic headache since her neurological evaluations were all normal. Her SLEDAI-2K score was only 1 when excluding the headache, indicating stable SLE. Based on her previous medical history such as recurrent respiratory infections and low IgM level, we considered other conditions that may also cause SLE phenotypes, especially inborn errors of immunity. Therefore, we performed whole exome sequencing (WES) and identified a homozygous pathogenic splice-disrupting variant in *SBDS* (NM_016038, c.258 + 2 T > C). Sanger sequencing confirmed that her parents were both heterozygous carriers (Fig. 1g). We have carefully looked for other monogenic SLE causes and quantified the 6 inteferon-stimulated genes (ISGs) in the blood (*IFIT1*, *IFI27*, *IFI44L*, *ISG15*, *SIGLEC1*, *RSAD2*) by quantitative polymerase chain reaction (qPCR). Her interferon score was normal with a median fold change of 1.26, and no other monogenic causes for SLE were found. Further investigations on classical SDS clinical phenotypes were performed. Abdominal CT showed shrunken pancreas with fat infiltration (Fig. 1d). Laboratory examinations revealed low amylase and positive Sudan III staining of the stool. Her bone marrow biopsy including morphology and cellularity was normal. X-ray showed osteoporosis of the right hip joint and avascular necrosis of the left femoral head (Fig. 1e), with no evidence of metaphyseal dysplasia. She was eventually diagnosed as SDS manifested with SLE. During the evaluation period, her headache symptoms resolved spontaneously. Therefore, we recommended continuation of prednisone and MMF therapy. She was then followed at local hospital after discharge due to the convenience reason.

At the age of 19, she developed numbness and tenderness of the lower limb and fingertips, irritability, and newly-onset oral ulcers. Brain MRI showed multiple abnormal signals in the brainstem, left hippocampus, right temporal lobe, and right frontal lobe. Cerebrospinal fluid pressure was 240 mmHg. The local hospital considered SLE flare and started to administer CTX in despite of negative autoantibodies. This time, the symptoms were not relieved and she gradually developed intermittent abdominal pain, dizziness, blurred vision, and Reynaud’s phenomenon.

At the age of 21, a routine follow-up of her complete blood panel showed anemia and leukopenia with blasts and Auer rods in her blood smear. Her platelet count was normal. A bone marrow aspirate was ordered which returned in support of acute myeloid leukemia (AML-M2) (Fig. 1f). She is currently awaiting a bone marrow transplant due to poor response to chemotherapy. Figure 1h shows the timeline of clinical symptoms and therapies.

In this case, we presented an SDS patient initially presented with SLE as her primary clinical phenotype. To our knowledge, this is the first case report of SLE in patients with SDS. Previous studies showed that SDS is an autosomal recessive disease with defective hematopoietic progenitors and impaired marrow microenvironment through the Fas pathway [1]. Predominantly, SDS presents with hematologic abnormalities, exocrine pancreatic insufficiency, and skeletal abnormalities. Other manifestations of SDS have been reported, involving endocrine, neuropsychiatric, and cardiovascular systems. MDS and AML are the most common complications of SDS, and MDS was reported to occur in approximately 15–20% of SDS patients with a high risk of AML transformation.

Some studies also reported immunological manifestations in SDS including hemophagocytic lymphohistiocytosis, autoimmune-like liver disease, arthritis, chronic recurrent multifocal osteomyelitis (CRMO), scleroderma, and inflammatory bowel disease [2–4]. The mechanisms of the immunological abnormalities in SDS remain unclear.

Our patient is a special SDS case, as she had typical clinical features of SLE. We also propose that mutations in the *SBDS* gene may be a novel genetic origin of monogenic lupus once more mechanistic data show up. To date, around 50 monogenic causes of SLE or lupus-like phenotype have been described, including defects of complement components, type I interferon (IFN) signaling, self-tolerance, Fas/FasL, and other pathways [5]. The tumor necrosis factor (TNF) superfamily ligand Fas ligand (FasL) plays a role in SLE pathogenesis through dysregulated apoptosis and/or inflammation. Previous study [1] reported that SBDS-deficient cells overexpress Fas and undergo accelerated spontaneous and Fas-mediated apoptosis. We hypothesize that the *SBDS*-associated lupus may be mediated through dysregulated FAS-FASL-associated cell death, triggering enhanced inflammation with release of damage-associated molecular patterns (DAMPs). However, data is still sparse to date, and more deep immunological investigations of more SDS patients with immune disorders are warranted.

## Data Availability

The datasets generated during and/or analyzed during the current study are available from the corresponding author on reasonable request.
